# Analysis of Multi-Antenna GNSS Receiver Performance under Jamming Attacks

**DOI:** 10.3390/s16111937

**Published:** 2016-11-17

**Authors:** Niranjana Vagle, Ali Broumandan, Gérard Lachapelle

**Affiliations:** PLAN Group, Department of Geomatics Engineering, Schulich School of Engineering, University of Calgary, 2500 University Drive N. W., Calgary, AB T2N 1N4, Canada; abrouman@ucalgary.ca (A.B.); gerard.lachapelle@ucalgary.ca (G.L.)

**Keywords:** GNSS, array processing, measurement distortions, carrier phase positioning

## Abstract

Although antenna array-based Global Navigation Satellite System (GNSS) receivers can be used to mitigate both narrowband and wideband electronic interference sources, measurement distortions induced by array processing methods are not suitable for high precision applications. The measurement distortions have an adverse effect on the carrier phase ambiguity resolution, affecting the navigation solution. Depending on the array attitude information availability and calibration parameters, different spatial processing methods can be implemented although they distort carrier phase measurements in some cases. This paper provides a detailed investigation of the effect of different array processing techniques on array-based GNSS receiver measurements and navigation performance. The main novelty of the paper is to provide a thorough analysis of array-based GNSS receivers employing different beamforming techniques from tracking to navigation solution. Two beamforming techniques, namely Power Minimization (PM) and Minimum Power Distortionless Response (MPDR), are being investigated. In the tracking domain, the carrier Doppler, Phase Lock Indicator (PLI), and Carrier-to-Noise Ratio (C/N_0_) are analyzed. Pseudorange and carrier phase measurement distortions and carrier phase position performance are also evaluated. Performance analyses results from simulated GNSS signals and field tests are provided.

## 1. Introduction

Applications of antenna arrays for mitigating different types of Global Navigation Satellite System (GNSS) interference have been widely studied [1,2,3,4,5,6,7,8,9,10,11]. Unlike other wireless applications, the quality of GNSS measurements after interference mitigation is important for high precision applications. However, antenna array processing induces distortions in code and carrier phase measurements in the presence of electronic interference [12,13,14,15,16,17,18,19,20,21,22,23]. The distortions in GNSS measurements caused by antenna array processing have been studied previously; for instance, [12,20] demonstrated distortions in carrier phase measurements with actual data and showed the dependency of distortions on satellite direction. In [13], code phase biases on the order of one to two metres using Monte-Carlo simulations in the presence of interference and mutual coupling were observed using the Minimum Variance Distortionless Response (MVDR) technique. In [8], authors studied phase tracking loop performance for possible carrier phase distortions due to different array processing techniques such as minimum variance and MVDR; it was shown through simulations that the minimum variance or power minimization based beamformers suffer comparatively more phase distortion than a MVDR based technique. Global Positioning System (GPS) phase measurement distortions due to phase centre variations of individual antenna elements in an array are studied in [18,19]. It has been shown that mutual coupling between antennas also causes phase distortions of a few centimetres; these are not tolerable for precise applications as they render carrier phase ambiguity resolution more difficult to achieve. In addition, code phase measurement biases due to the phase delay and group delay of antenna elements were observed. Although code phase and carrier phase measurement distortions were reported, a detailed analysis of these and navigation solution quality for high precision applications was not completed. In this research, the performance of a multi-antenna GNSS receiver is evaluated for different array processing techniques in terms of carrier tracking, measurement, and position distortions. Both narrow band and wide band interference sources are considered. Two array processing techniques, namely PM beamformer, which does not take Angle-of-Arrival (AOA) information of satellites into consideration, and MPDR beamformer, which uses the AOA of satellites [24], are considered. The methodology for evaluating possible measurement distortions is described and followed by a discussion of the results using simulated and actual data. Performance evaluations are carried out using GPS L1 Coarse/Acquisition (C/A) signals.

## 2. Methodology

This section describes GNSS and interference signal simulations, the multi-antenna signal processing method, and the methodology to characterize the code and carrier phase measurement distortions used in the sequel.

### 2.1. Signal and System Model

**Figure 1:**
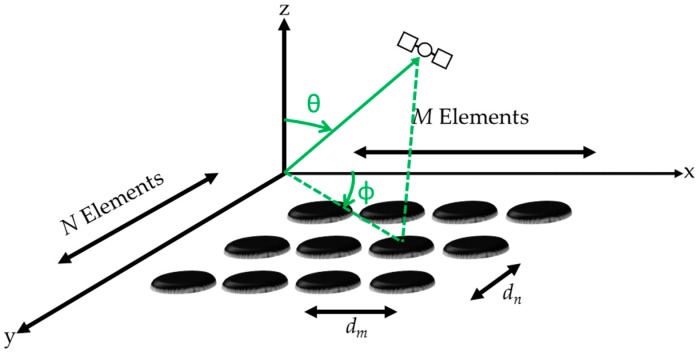
Rectangular array structure with *M × N* elements.

A rectangular planar array planar is used herein. Consider the case of an *M* × *N* element uniform rectangular array. The elements are lying in the *x-y* plane and are equally spaced by *d_m_* in the *x*-direction and *d_n_* in the *y*-direction as shown in Figure 1.

After down-conversion and sampling, the digitized signal received at the (*m*, *n*)th antenna element can be expressed as

(1) $$x_{m,n}\left(t\right)=\overset{K}{\underset{k=1}{\sum }}s_{k}\left(t\right)e^{j\frac{2\pi }{\lambda }\left[\left(m-1\right)d_{m}\sin\left(\theta_{k}\right)\sin\left(\phi_{k}\right)+\left(n-1\right)d_{n}\sin\left(\theta_{k}\right)\cos\left(\phi_{k}\right)\right]}\;\;+\overset{J}{\underset{j=1}{\sum }}j_{j}\left(t\right)e^{j\frac{2\pi }{\lambda }\left[\left(m-1\right)d_{m}\sin\left(\theta_{j}\right)\sin\left(\phi_{j}\right)+\left(n-1\right)d_{n}\sin\left(\theta_{j}\right)\cos\left(\phi_{j}\right)\right]}+\eta_{m,n}\left(t\right)$$

where $s_{k}\left(t\right)$ is the *k*th satellite signal component, $j_{j}\left(t\right)$ is the *j*th electronic interference, λ refers to the wavelength of the signal, $\left(\theta_{k},\phi_{k}\right)$ is the elevation and azimuth angle of the *k*th satellite, $\left(\theta_{j},\phi_{j}\right)$ is the elevation and azimuth angle of the electronic interference signal, *K* is the number of Pseudo Random Numbers (PRNs), *J* is the number of electronic interference signals, and $\eta_{m,n}\left(t\right)$ is the additive spatially white noise of the (*m*, *n*)th antenna element.

The impinged signals from all the antenna elements can be represented in matrix form as

(2) x=As+Bj+η

where x is the *MN* × 1 received signal vector, η is the *MN* × 1 noise vector, s is the *K* × 1 satellite signal vector, and j is the *J* × 1 signal vector affected by electronic interference; these vectors are given by

(3) $$x={\left[x_{1,1}\left(t\right),x_{2,1}\left(t\right),\ldots ,\;x_{M,1}\left(t\right),x_{1,2}\left(t\right),\ldots ,\;x_{M,N}\left(t\right)\right]}^{T}$$

(4) $$s={\left[s_{1}\left(t\right),s_{2}\left(t\right),\ldots ,s_{K}\left(t\right)\right]}^{T}$$

(5) $$j={\left[j_{1}\left(t\right),j_{2}\left(t\right),\ldots ,j_{J}\left(t\right)\right]}^{T}$$

(6) $$\eta ={\left[\eta_{1,1}\left(t\right),\eta_{2,1}\left(t\right),\ldots \eta_{M,1}\left(t\right),\eta_{1,2}\left(t\right),\ldots \eta_{M,N}\left(t\right)\right]}^{T}$$

The steering matrices A and B are of dimensions *MN* × *K* and *MN* × *J* respectively and are given by

(7) $$A={\left[a_{1},a_{2},\;\ldots ,\;a_{k}\right]}^{T}$$

(8) $$B={\left[b_{1},b_{2},\;\ldots ,\;b_{j}\right]}^{T}$$

where $a_{k}$ is the *MN* × 1 steering vector of the *k*th satellite signal component coming from direction $\left(\theta_{k},\phi_{k}\right)$ and $b_{j}$ is the *MN* × 1 steering vector of the *j*th electronic interference signal coming from direction $\left(\theta_{j},\phi_{j}\right)$. Both $a_{k}$ and $b_{j}$ have a similar structure, hence only $a_{k}$ is defined and is given by

(9) $$a_{k}={\left[c_{k}^{T},\gamma_{k}c_{k}^{T},\;\ldots ,\;\gamma_{k}^{N-1}c_{k}^{T}\right]}^{T}$$

(10) $$c_{k}={\left[1,\beta_{k},\;\ldots ,\;\beta_{k}^{M-1}\right]}^{T}$$

(11) $$\gamma_{k}=e^{j\frac{2\pi }{\lambda }\left[d_{n}\sin\left(\theta_{k}\right)\cos\left(\phi_{k}\right)\right]}$$

(12) $$\beta_{k}=e^{j\frac{2\pi }{\lambda }\left[d_{m}\sin\left(\theta_{k}\right)\sin\left(\phi_{k}\right)\right]}$$

### 2.2. Multi-Antenna Signal Simulations and Processing

The multi-antenna GNSS signal simulator developed in [25] was used to generate multi-antenna signals with different types of interference. For the simulations, it was assumed that antenna elements are identical and there is no mutual coupling. A static user scenario was considered and the interference source was also assumed to be static. The simulated signals are processed using a modified version of the GSNRx^TM^ software receiver [26] to generate code and carrier phase measurements. The software receiver was modified to incorporate multi-antenna processing with Power Minimization (PM) and MPDR processing techniques.

#### 2.2.1. Power Minimization (PM)

**Figure 2:**
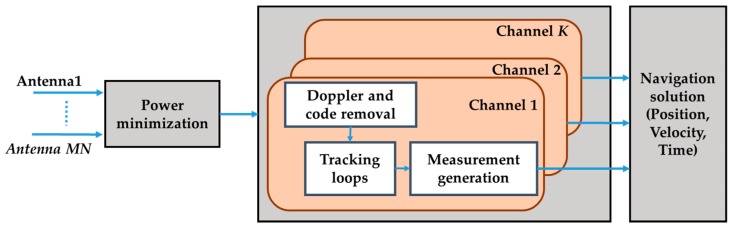
Multi-antenna processing using PM beamforming.

The PM approach was implemented in the pre-correlation stage with the receiver architecture shown in Figure 2. The covariance matrix of the received signal can be obtained as

(13) $$R_{xx}=\frac{1}{T}\overset{T}{\underset{1}{\sum }}xx^{H}$$

where T is the number of temporal Intermediate Frequency (IF) samples and $R_{xx}$ is of dimension *MN × MN*.

The optimum weight vector is given by [27]

(14) $$w_{\mathrm{PM}}=\frac{R_{xx}^{-1}q}{q^{H}R_{xx}^{-1}q}$$

where q is the constraint vector with dimension *MN* × 1 and given by

(15) $$q={\left[1,\;0,\;0,\;\ldots ,\;0\right]}^{T}$$

#### 2.2.2. Minimum Power Distortionless Response (MPDR)

**Figure 3:**
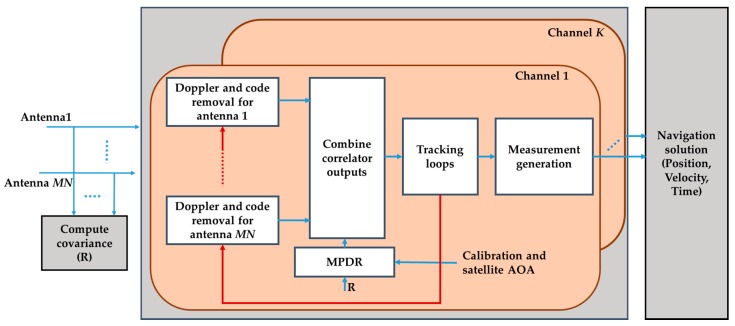
Multi-antenna processing approach using MPDR beamforming.

MPDR was performed at the post-correlation stage with the MPDR receiver architecture shown in Figure 3. IF signals from all the antennas are fed to each tracking channel. In each tracking channel, the code and carrier replica signals from the beamformed data are used to de-spread other antenna signals in order to maintain relative phase differences between the antenna elements. The optimum weight vector for the MPDR beamformer for the *k*th satellite is given by [24]

(16) $$w_{\mathrm{PM}}=\frac{R_{xx}^{-1}a_{k}}{a_{k}^{H}R_{xx}^{-1}a_{k}}$$

The covariance matrix $R_{xx}$ was computed at the pre-correlation stage because at this stage, interference power might be reduced due to the correlation process affecting beamformer performance. The combined correlator outputs will be used to generate code and carrier phase measurements.

### 2.3. Measurement Distortions Analysis

After interference suppression, the main goal is to characterize distortions in terms of mean and standard deviations in the code and carrier phase measurements from the beamforming process in the presence of interference. A differential technique is used to evaluate the measurement distortions. Measurements from one of the array antennas with clean data without interference acts as a reference channel. Single differencing between reference antenna measurements (with clean data) and those from the beamformed data (in the presence of interference) acts as a zero-baseline test. The resulting single difference should ideally have a zero mean if there are no distortions in the measurements from the antenna array processing. Measurement distortions refer to the distortions caused by the multi-antenna GNSS receiver process in the presence of interference. 

#### 2.3.1. Pseudorange Measurements Analysis

The pseudorange measurement of the *k*th satellite after beamforming in the presence of interference can be expressed as

(17) $$P_{array}^{k}=\rho^{k}+d\rho^{k}+c\left(dt^{k}-dT\right)+d_{iono}^{k}+d_{tropo}^{k}+\rho_{bias}^{k}+\varepsilon_{\rho }^{k}$$

where $\rho^{k}$ is the true range, $d\rho^{k}$ is the orbital error, $dt^{k}$ and dT are the satellite and receiver clock errors, $d_{iono}^{k}$ is the ionosphere error, $d_{tropo}^{k}$ is the tropospheric delay, $\varepsilon_{\rho }^{k}$ is the receiver code noise, and $\rho_{bias}^{k}$ is the possible bias introduced by antenna array processing. 

The pseudorange from the *k*th satellite for the reference antenna without interference can be expressed as

(18) $$P_{ref}^{k}=\rho^{k}+d\rho^{k}+c\left(dt^{k}-dT\right)+d_{iono}^{k}+d_{tropo}^{k}+\varepsilon_{\rho }^{k}$$

The single difference between the pseudorange measurements obtained from the reference antenna and after beamforming is

(19) $$\Delta P^{k}=P_{ref}^{k}-P_{array}^{k}=\rho_{bias}^{k}+\varepsilon_{\Delta \rho }^{k}$$

where $\varepsilon_{\Delta \rho }^{k}$ is the single differenced receiver code noise.

Since this single differencing acts as a zero-baseline, the atmospheric error and multipath cancel out. The code noise standard deviation depends on receiver tracking strategies such as chip spacing and the discriminators used and it should ideally be zero mean. Hence, one can estimate the pseudorange bias induced by the antenna array processing in the presence of interference. It is assumed that the residual multipath effect is small compared to the antenna array induced biases. In the simulations, GPS signals were simulated in multipath free conditions. In the case of actual data collection, a low multipath environment was selected.

#### 2.3.2. Carrier Phase Measurements Analysis

Similar to pseudorange measurement bias estimation, the carrier phase measurement bias can also be estimated by performing single differencing between the reference antenna and beamformed carrier phase measurements. 

The carrier phase measurements of the *k*th satellite after beamforming in the presence of interference can be expressed as

(20) $$\phi_{array}^{k}=\rho^{k}+d\rho^{k}+c\left(dt^{k}-dT\right)+\lambda N^{k}-d_{iono}^{k}+d_{tropo}^{k}+\phi_{bias}^{k}+\varepsilon_{\phi }^{k}$$

where $\varepsilon_{\phi }^{k}$ is the receiver carrier phase noise, $N^{k}$ is the integer ambiguity, and $\phi_{bias}^{k}$ is the possible carrier phase bias introduced by antenna array processing.

The carrier phase measurement from the *k*th satellite for the reference antenna without interference can be expressed as

(21) $$\phi_{ref}^{k}=\rho^{k}+d\rho^{k}+c\left(dt^{k}-dT\right)+\lambda N^{k}-d_{iono}^{k}+d_{tropo}^{k}+\varepsilon_{\phi }^{k}$$

The single difference between the pseudorange measurements obtained from the reference antenna and after beamforming is

(22) $$\Delta \phi^{k}=\phi_{ref}^{k}-\phi_{array}^{k}=\phi_{bias}^{k}+\varepsilon_{\Delta \phi }^{k}$$

where $\varepsilon_{\Delta \phi }^{k}$ is the single differenced receiver carrier phase noise.

After single differencing, atmospheric errors cancel out. There is no receiver clock error since a single receiver is used. Integer ambiguity is also removed since a coherent-phase multi-channel front-end using a single oscillator is used which makes receiver to initialize ambiguity to the same value for the single channel and multi-antenna receiver process. As in the pseudorange case, carrier phase noise is ideally zero mean and therefore any bias observed using single differencing can be attributed to array processing in the presence of interference.

As this research focuses on high precision applications, antennas with good phase centre stability and low mutual coupling are considered. Hence, the effect of phase centre and mutual coupling are not considered in the pseudorange and carrier phase measurement formulations (Equations (17)–(22)).

## 3. Simulation Results and Discussion

The effect of different types of interference sources and array processing approaches are now studied through simulations. Simulations were carried out considering different interference types. In order to evaluate the effect of different kinds of interference and different beamforming methods, it is assumed that only one interference source is present at the time in each simulation scenario. The interference sources considered are Continuous Wave (CW) interference, chirp interference, and band-limited additive white Gaussian noise. The effect of these interference sources on GNSS receiver operation differs due to their spectral characteristics. Therefore, it is of interest to check the performance of the array based receiver under different interference types. Even though the power and the number of interference sources eventually affect beamformer performance, the main goal here is to compare PM and MPDR beamformers for high precision applications. 

**Figure 4:**
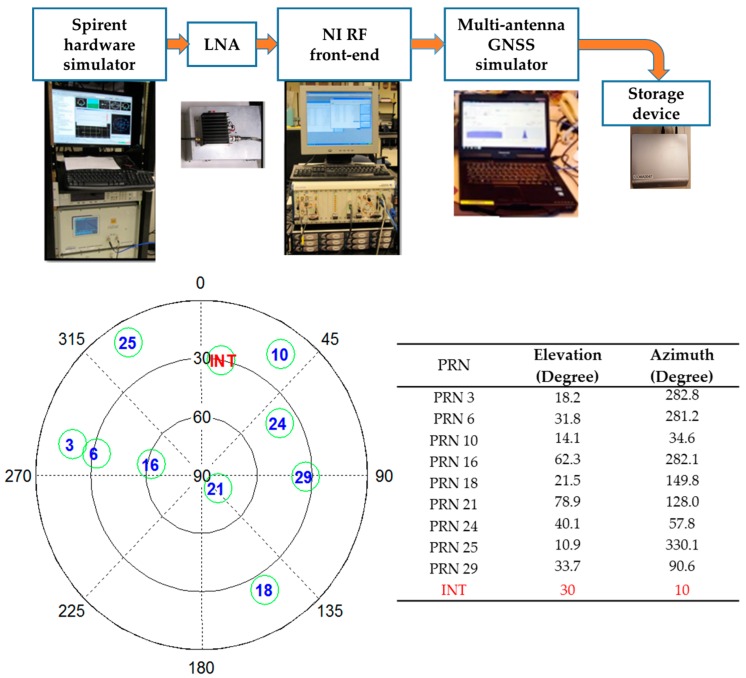
Multi-antenna signal simulation set-up and sky plot of the simulated GPS satellites and interference (INT) source.

The input IF data file for the multi-antenna software simulator was collected using a Spirent hardware GPS simulator [28] by disabling multipath and atmospheric errors. A static user scenario was used for collecting IF data samples at a 5 MHz sampling rate. The multi-antenna GPS signals were generated using the multi-antenna signal simulator and interference was added later to the clean IF samples. The six-element rectangular antenna array described earlier was considered for the simulations. The interference source was assumed to be static and was added after 15 s in order for the receiver to acquire and track the signals (for the post-correlation beamforming). The multi-antenna signal simulation method and sky plot of the simulated GPS satellites and interference source direction are shown in Figure 4. The characteristics of the interference source are given in Table 1. In all the simulation scenarios, the interference source direction was the same. The C/N_0_ values for all PRNs were set to be the same for all the antenna elements (48 dB-Hz).

The simulated signals were processed using GSNRx^TM^ with the architectures shown in Figure 2 and Figure 3 to generate tracking and measurement parameters. Here, PM was performed from the beginning of the dataset as it was done in the pre-correlation stage. In the case of the MPDR beamformer, the receiver was made to operate with a single antenna for the initial 10 s, after which beamforming was performed before the occurrence of interference. This was done to ensure that the receiver operates in beamforming mode when interference affects the IF samples. In both PM and MPDR approaches, correlator spacing of 0.4 between early and late arms and non-coherent early-minus late discriminators were used. Carrier tracking was performed using a third order Phase Locked Loop (PLL) with a 15 Hz bandwidth.

### 3.1. Tracking Domain Analysis

In this section, tracking performance in terms of carrier Doppler estimation, C/N_0_ and Phase Lock Indicator (PLI) is evaluated. The C/N_0_ is computed using narrow band power and wide band power of the prompt correlation values and PLI is computed using prompt in-phase and quadrature-phase values [29]. Initially, the signal affected by interference from the reference antenna of the array was processed and the corresponding results are discussed in subsequent sections. Since PRN 6 and PRN 16 are more affected by CW interference, these two PRNs are used to analyze tracking performance of the array-based receiver. 

**Figure 5:**
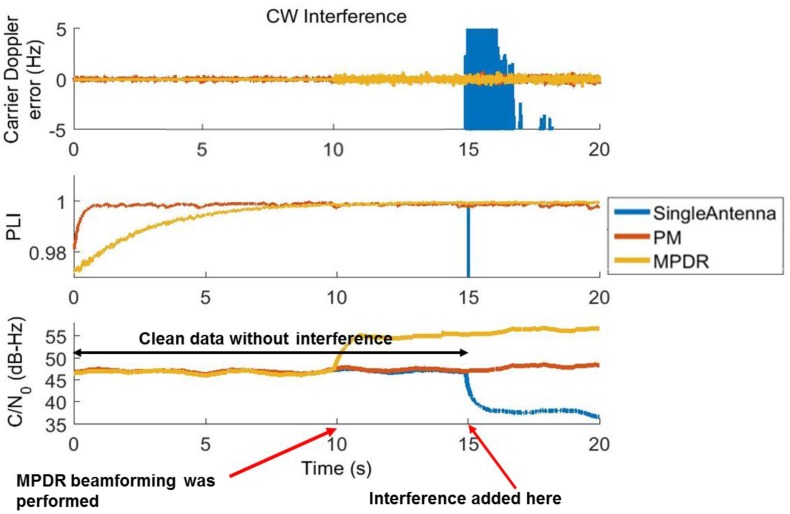
Tracking performance of PRN 6 with CW interference.

The tracking outputs of PRN 6 for the single antenna case affected by CW interference after beamforming for the first 20 s are shown in Figure 5 where ‘Single Antenna’ corresponds to the tracking outputs of the reference antenna of the array in the presence of interference. For the initial 15 s, there was no interference; as mentioned earlier, PM was performed from the beginning of the data and MPDR was performed after 10 s. 

Let us first consider the initial 15 s of data without interference. After MPDR beamforming, a C/N_0_ gain of 7.5 dB is observed. As six antenna elements were used, a gain of 7.7 dB [10 × log_10_(number of antennas)] is expected and one can observe the same with MPDR beamforming. With PM, a gain of 1.5 dB is observed; in this case, since there is no constraint on for the satellite signal AOA, the gain depends on the beampattern, which in turn depends on the interference direction and array geometry. 

Now consider the data affected by interference (after 15 s) in Figure 5. As soon as interference is injected, the C/N_0_ values of the single antenna drops by about 8 dB. However, the same C/N_0_ as that without interference was maintained through MPDR and PM methods. This ensured successful mitigation of the interference. If one considers the C/N_0_ gain in the presence of interference, MPDR was able to deliver nearly 15.5 dB with respect to the single antenna. The gain observed from PM was 9.5 dB. As shown in Figure 5, Doppler errors increase and PLI degrades for the single antenna case after interference. However, MPDR and PM were able to provide the same performance as that for the interference-free case. To compute the carrier Doppler errors shown in Figure 5, the Doppler values obtained from the reference antenna with clean data were used as reference.

**Figure 6:**
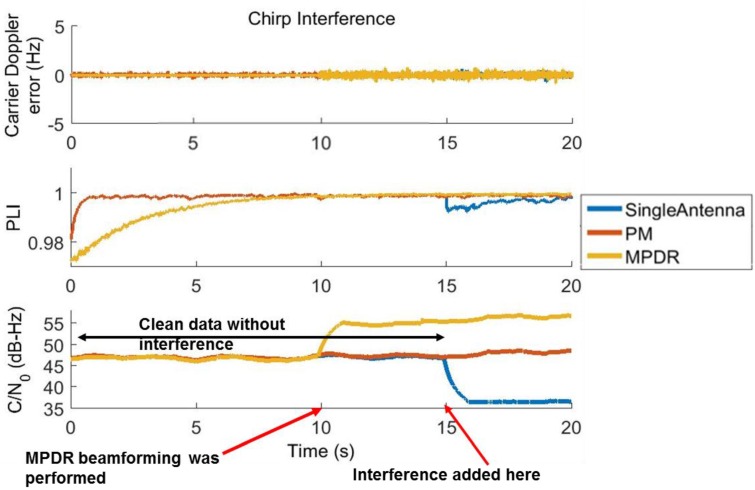
Carrier tracking performance of PRN 6 with chirp interference.

**Figure 7:**
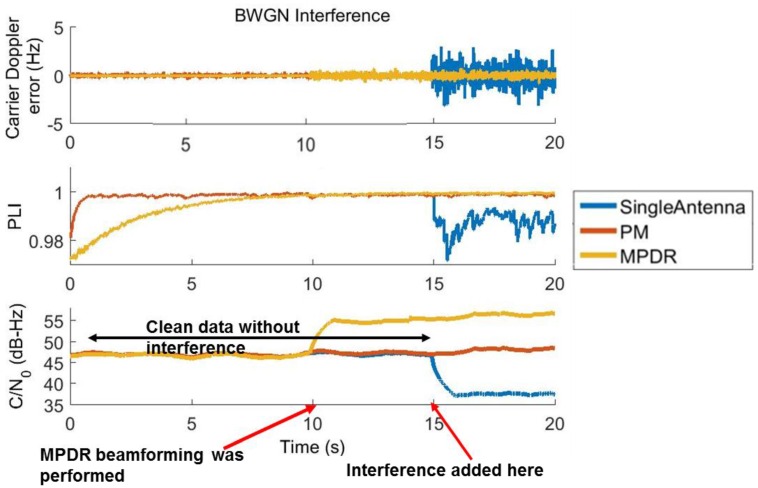
Carrier tracking performance of PRN 6 with Band limited white Gaussian noise.

The carrier tracking performance of PRN 6 for chirp and BWGN scenarios for the first 60 s are shown in Figure 6 and Figure 7. MPDR was able to provide 15.5 dB of gain even in the presence of chirp and BWGN interference. PM was able to provide 9.5 dB of gain in the presence of the same interference. The Doppler error variance is lower after beamforming; this can be clearly observed in Figure 7 in the presence of BWGN interference. The receiver was able to maintain PLI values of 0.99 after beamforming in all interference scenarios, indicating reliable tracking of the carrier phase. 

The performance results of different PRNs in terms of Root-Mean-Square (RMS) Doppler errors, C/N_0_ gain, and mean PLI for chirp interference are listed in Table 2. These values were computed using the tracking outputs in the presence of interference. For computing Root-Mean-Square Error (RMSE) Doppler errors, the Doppler values obtained from the reference antenna with clean data were used as true values. The tracking performance results of different PRNs in the presence of CW and BWGN jammers are provided in Appendix A for the CW jammer in Table A1 and those for BWGN in Table A2. It can be observed that compared to single antenna results, RMS Doppler errors are reduced for both beamformers for all simulation scenarios. MPDR RMS Doppler errors were lower by 0.1 Hz as compared to PM, a relative improvement of 25%. 

### 3.2. Measurement Domain Analysis

This section analyzes pseudorange and carrier phase measurement errors after beamforming. The measurement domain results in the presence of chirp interference are provided here. The measurement differencing technique described in the methodology section is used to obtain pseudorange and carrier phase measurement errors.

**Figure 8:**
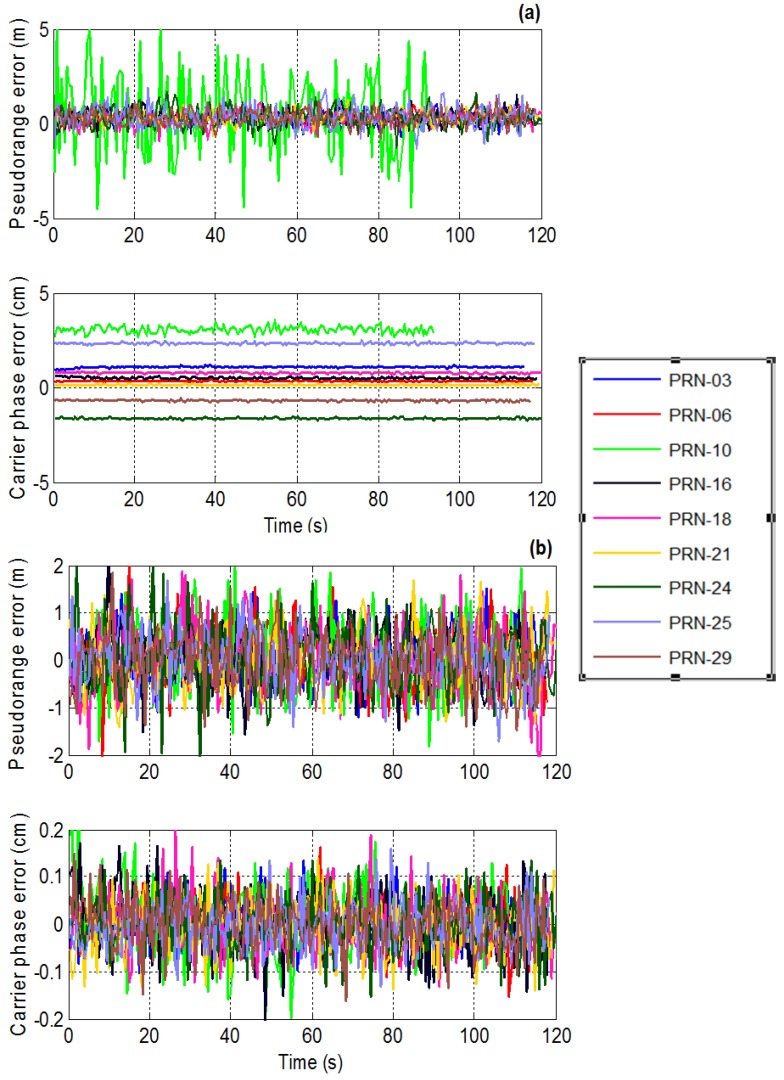
Time series plot of the pseudorange and carrier phase measurement errors using (**a**) PM; (**b**) MPDR.

A time series plot of the pseudorange and carrier phase measurement errors for both PM and MPDR are shown in Figure 8. The mean and standard deviations of the pseudorange errors for all the PRNs are provided in Table 3. First consider the results of PM. It can be seen from Table 3 that PM induces pseudorange biases of the order of 30 cm to 50 cm for different PRNs. It can also be observed that the biases observed are different for different PRNs. It can be seen from Figure 8 that PRN 10 distortions are higher than those of other PRNs. This is because the interference source AOA is close to that of PRN 10 as shown in the sky plot of Figure 4. Now consider the MPDR beamformer; it induces up to 4 cm of pseudorange bias, which is about the code phase measurement noise. For PRN 10, which is near the interference source, standard deviations of the measurement errors have increased compared to other PRNs. 

It can be observed that mean pseudorange bias values are higher for PM than MPDR. The mean and standard deviations of the carrier phase measurement errors for different PRNs are listed in Table 4. It can be observed from Figure 8 that PM induces different biases for different PRNs. The bias for a particular PRN remains the same for the entire test duration. This is because the interference source location and the array orientation were fixed and as such the beamformer weights were constant during the test. However, if the interference direction is changed or in the case of a dynamic user, the beamformer weights also change, different biases might be observed for the same PRN. A maximum bias value of 30 mm was observed in PRN 10 bias in the case of the PM beamformer. The carrier phase measurement biases for the MPDR beamformer are minimal as listed in Table 4. 

Similar observations were made with CW and BWGN jammers for code and carrier phase measurement distortions. The results corresponding to these jammers are provided in Table B1, Table B2, Table B3 and Table B4.

### 3.3. Position Domain Analysis

For the position domain analysis, the code and carrier phase measurements were processed using the RTKLib open source software [30]. The measurements generated using the reference antenna of the array without interference were used as the base observations for the RTK software. The remote observations were generated from the beamformed data affected by interference. This kind of processing acts like a zero-baseline and any distortion in the measurements affects the position solution. The open source software was used without any modifications. In the RTKLib, the continuous carrier phase ambiguity resolution mode was selected and forward-backward processing was performed.

**Figure 9:**
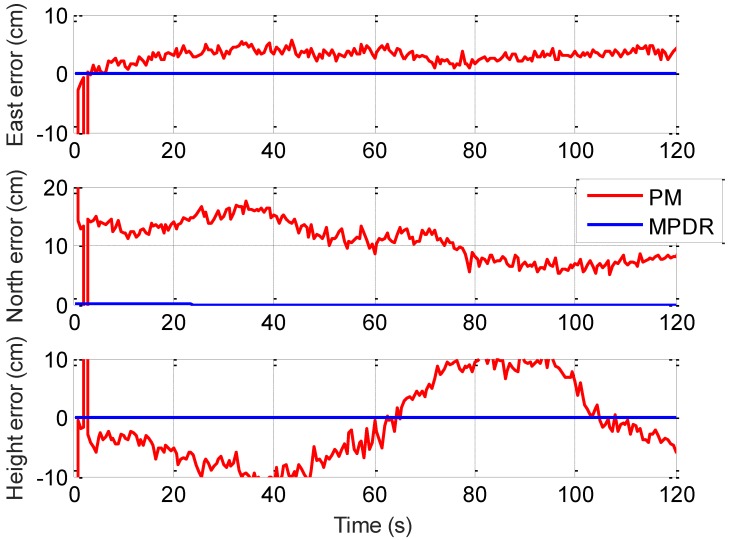
Carrier phase position errors with PM and MPDR in the presence of chirp interference (Processed using RTKLib open source software).

The east, north, and height position errors for PM and MPDR with chirp interference are shown in Figure 9. The RTK software outputs a quality flag for each solution update indicating a fixed or float solution. PM was not able to provide fixed solutions and position errors on the order of 10 cm occur. MPDR was able to provide fixed solutions and position errors were at the sub-millimetre level. The mean and standard deviations of the position errors observed for both beamformers are provided in Table 5. In the PM case, satellite directions are not considered in the optimization criterion and the antenna phase values are not added coherently. This will have adverse effect on the carrier phase measurements as discussed in the measurement analysis section. These biased measurements will affect carrier phase based positioning. It should be noted that with PM, different biases are observed for different PRNs and these bias values are a function of the interference and satellites AOA and cannot be calibrated beforehand. 

### 3.4. Effect of Interference AOA on Measurements

**Figure 10:**
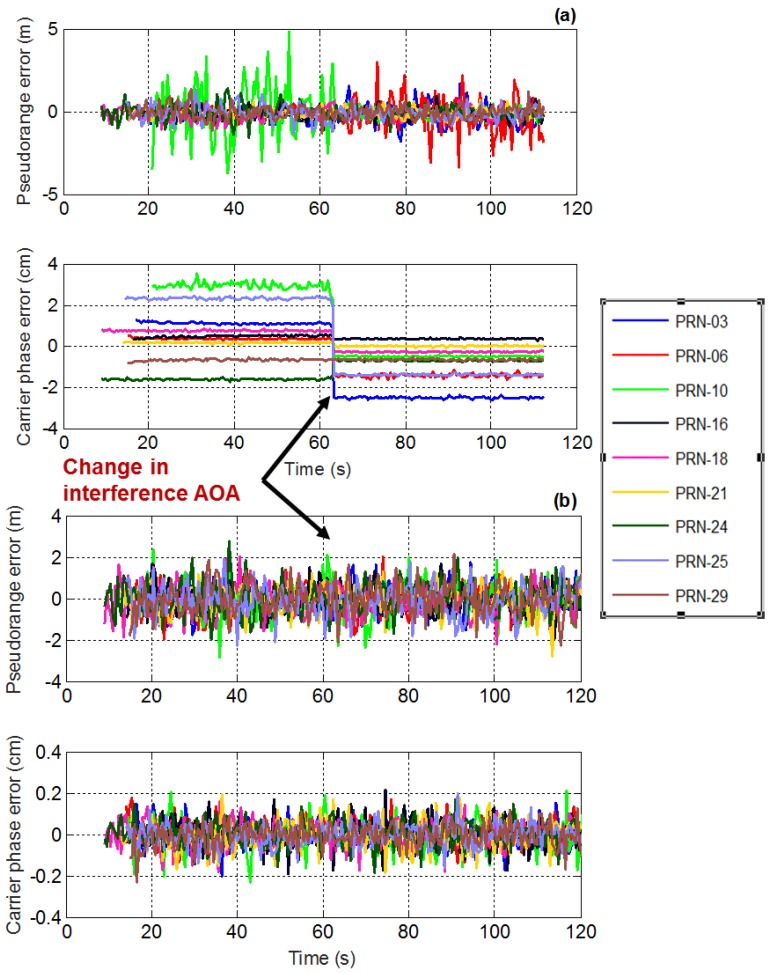
Effect of change in AOA of interference on pseudorange and carrier phase measurements for (**a**) PM; and (**b**) MPDR.

The relative direction between jammer and satellite signals plays a critical role in anti-jam applications using antenna array processing. The effect of changing the AOA of the interference source is therefore analyzed. It is assumed that only one chirp interference source is present. Time series plot of the pseudorange and carrier phase measurements in the presence of chirp interference for PM and MPDR are shown in Figure 10. The interference direction is changed after 65 s. For the initial 65 s, the interference is located at (elevation = 30°, azimuth = 10°) and then changed to (elevation = 30°, azimuth = 225°). It can be observed that the carrier phase biases observed in PM changes when the interference direction changes. However, MPDR beamformer does not induce any biases in the carrier phase measurements, irrespective of the AOA of the interference. For the initial 65 s, interference location was near PRN 10 and due to this, the standard deviation of the pseudorange measurements for PRN 10 was high. After 65 s, the interference direction was away from PRN 10 and the standard deviation of the pseudorange measurements decreased. 

## 4. Field-Test Results and Discussion

**Figure 11:**
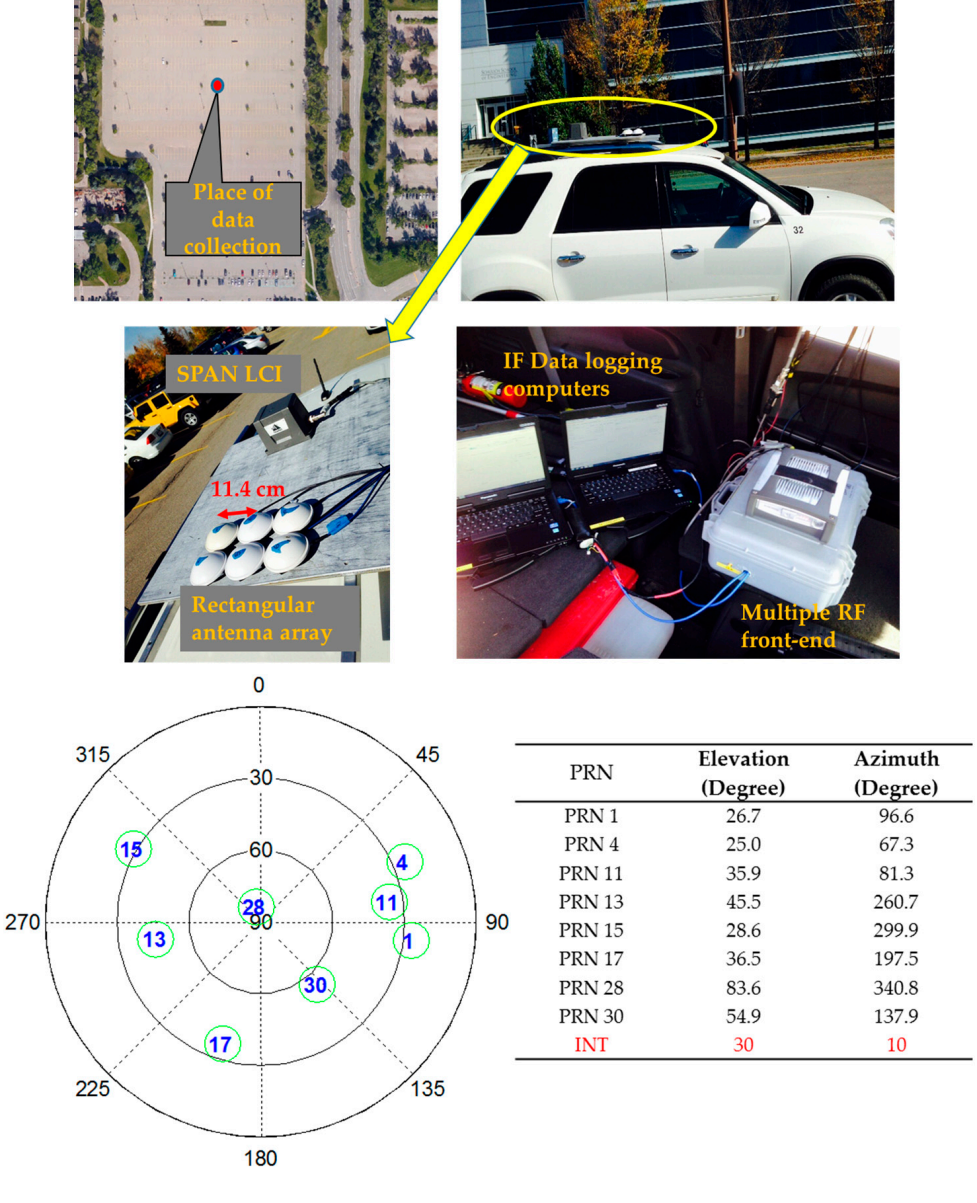
Field data collection setup and sky plot of visible GPS satellites.

This section describes field test results using PM and MPDR beamforming. Unlike the simulation case, the data collected in an open sky condition was subject to actual antenna array errors such as antenna phase centre instability, phase and gain mismatch between different receiver channels, and mutual coupling. A rectangular array with six antenna elements shown in Figure 11 was used for the data collection. Novatel 501 antenna elements [31] were used to construct the array. The setup used and sky plot of the visible GPS satellites are shown in Figure 11. A NovAtel Synchronous Position, Attitude and Navigation (SPAN) LCI mounted on the array platform was used to obtain the array attitude used for calibration and MPDR beamforming. A Fraunhofer multi-antenna RF front-end was used to collect IF samples from all the six antennas simultaneously in an open sky environment and low multipath conditions. The initial few seconds of the data were used to perform antenna array calibration. Interference was added to the data through a MATLAB^TM^ software script. Although interference was added later to the actual data as in the case of simulations, the difference between these simulations and field tests is the calibration of an actual array and mutual coupling which might affect the performance of the beamformer. The interference source is assumed to be static with a jammer-to-signal power of 30 dB. As beamformer performance was similar for different types of jammers, results corresponding to the chip jammer are provided in the field test results.

The collected data without interference and after adding interference were processed with GSNRx^TM^ to generate tracking parameters and measurements. A correlator spacing of 0.2 between the early and late arms and non-coherent early-minus late discriminator was used. Carrier tracking was performed using a third order PLL with a 15 Hz bandwidth.

### 4.1. Tracking Domain Analysis

**Figure 12:**
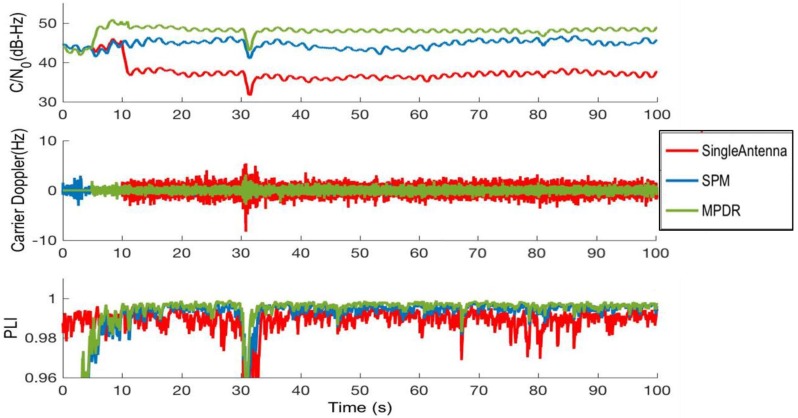
Tracking performance of PRN 4 with PM and MPDR using actual data with chirp interference.

The PM beamforming was performed from the beginning of the data and MPDR was performed after 10 s. The interference was added to the data after 15 s. Tracking performance of the low elevation PRN 4 is now considered. The tracking performance is shown in Figure 12. MPDR beamforming applied on clean data from 10 to 15 s showed nearly 6 dB of gain with respect to the single antenna. In the presence of interference, MPDR was able to maintain the gain. However, the gain obtained was higher in the presence of interference and was observed to be around 12 dB with respect to the single antenna case. The C/N_0_ gain observed with PM in the presence of interference was about 7 dB. The carrier Doppler errors were computed by taking the difference between the reference antenna Doppler with clean data without interference and beamformed data in the presence of interference. The Doppler RMS errors, PLI, and C/N_0_ gain for different PRNs are shown in Table 6. These values are computed from the tracking results in the presence of interference. For all the PRNs, PLL was able to maintain lock even in the presence of interference. 

### 4.2. Measurement Domain Analysis

**Figure 13:**
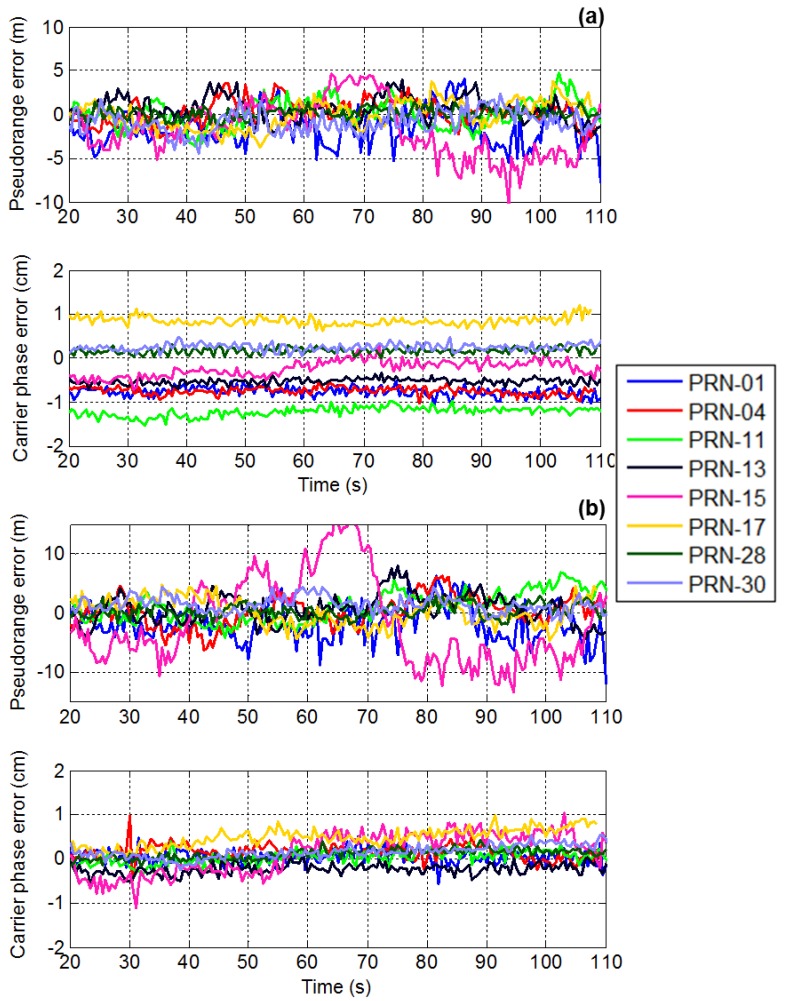
Pseudorange and carrier phase measurements with actual data with chirp interference for (**a**) PM; and (**b**) MPDR.

The measurements generated by the interference free reference antenna data acts as reference measurements in this case. Differences between these measurements and those generated after beamforming were compared for PM and MPDR. The resulting time series plot of the pseudorange and carrier phase measurement errors are shown in Figure 13 and the corresponding statistics are provided in Table 7 and Table 8. Pseudorange biases up to 2 m were observed in both PM and MPDR beamformers. The biases observed are different for different PRNs. Carrier phase biases are minimal (less than 1 mm) with the MPDR beamformer for all PRNs, which agrees with the simulation results. With PM, different biases were observed for different PRNs as seen from Figure 13a and they reach 1.1 cm for some PRNs. The results with PM also agree with simulation results where different biases were observed for different PRNs.

### 4.3. Position Domain Analysis

**Figure 14:**
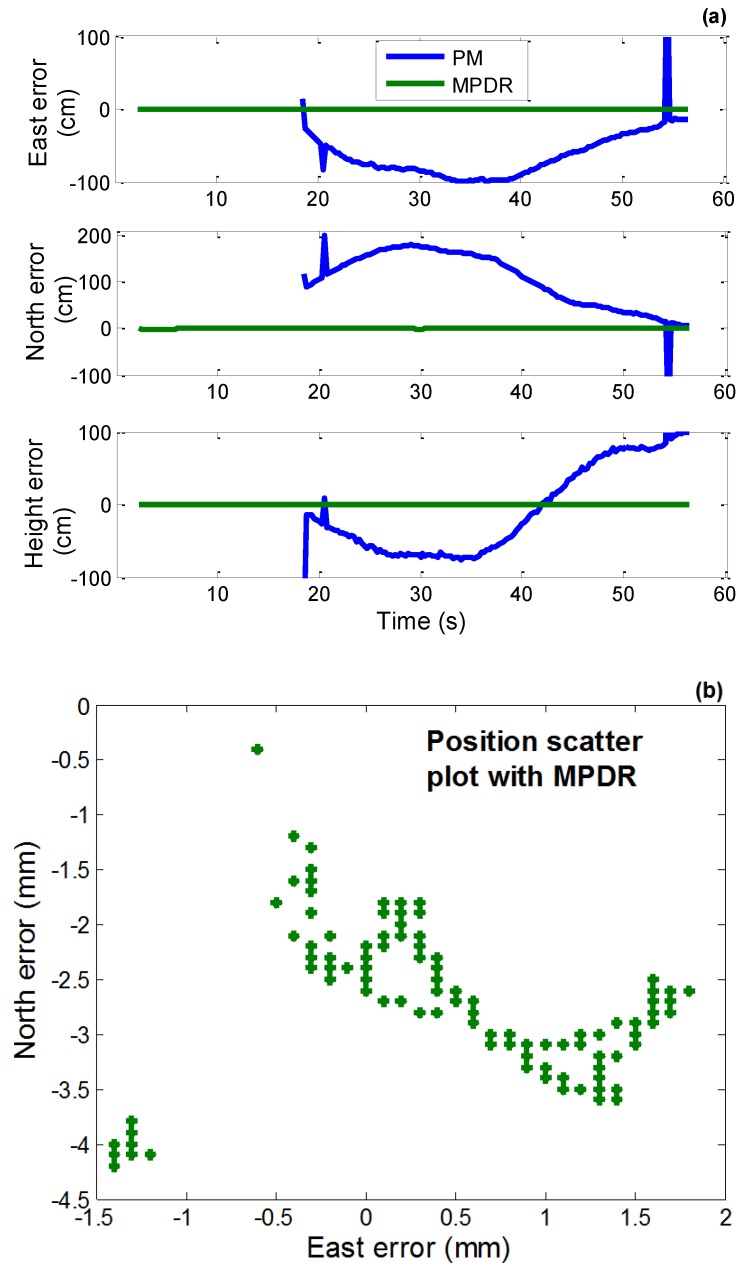
(**a**) Carrier phase based position errors with actual data with chirp interference using PM and MPDR; (**b**) East-north position errors scatter plot with MPDR.

Carrier phase based positioning was performed using RTKLib open source software. The position errors using PM and MPDR are shown in Figure 14a and the east-north position errors scatter plot using MPDR is shown in Figure 14b. The error statistics are shown in Table 9. MPDR is able to provide fixed solutions. However, PM was not able to provide fixed solution and errors up to 1 m were observed. 

## 5. Conclusions

The performance of a multi-antenna GNSS receiver under narrowband and wideband jamming conditions was investigated for high precision applications. Different types of electronic interference sources were considered for performance evaluations in terms of tracking, measurement, and position distortions for PM and MPDR beamformers. A number of GPS signal simulations were performed to evaluate measurement distortions. It was observed that MPDR did not induce measurement distortions in the simulations. With actual data, carrier phase measurement biases were minimal and pseudorange measurement errors up to 2 m were observed using MPDR. However, with PM, pseudorange biases up 50 cm were observed in the simulations and up to 2 m with actual data. Carrier phase measurement biases up to few cm were observed with PM in both simulations and actual data processing. The biases observed were different for different PRNs. Carrier phase positioning performance with MPDR was better than PM; the latter was unable to provide fixed carrier phase ambiguity solutions. With PM, position errors up to 10 cm were observed in simulations and up to 1 m with actual data. 

## Figures and Tables

**Table 1:** Interference sources considered in the simulations and their characteristics.

Simulation Scenarios	Interference Type	Interference Characteristics
Scenario 1	CW interference	The frequency of the CW interference is 500 Hz away from IF (420 kHz). Jammer-to-signal power is assumed to be 30 dB.
Scenario 2	Chirp interference	Chirp signal bandwidth is 11.8 MHz with an interval of 11.7 μs. These values are basically chosen from commercially available in-car jammers. Jammer-to-signal power is 30 dB.
Scenario 3	Band limited white Gaussian noise	White Gaussian noise is generated through the MATLAB^TM^ awgn() function with a bandwidth of 5 MHz and jammer-to-signal power of 30 dB.

**Table 2:** Tracking performance of different PRNs in the presence of chirp interference.

PRN	Single Antenna			PM			MPDR		
	RMSE Doppler (Hz)	PLI	C/N_0_ (dB-Hz)	RMSE Doppler (Hz)	PLI	C/N_0_ (dB-Hz)	RMSE Doppler (Hz)	PLI	C/N_0_ (dB-Hz)
PRN 3	0.45	0.99	36.6	0.32	0.99	48.9	0.26	0.99	54.9
PRN 6	0.40	0.99	36.6	0.36	0.99	48.0	0.28	0.99	54.7
PRN 10	0.46	0.99	36.7	0.78	0.99	45.8	0.36	0.99	54.4
PRN 16	0.74	0.99	36.7	0.61	0.99	45.9	0.51	0.99	54.4
PRN 18	0.43	0.99	36.7	0.40	0.99	47.3	0.35	0.99	54.4
PRN 21	0.42	0.99	36.7	0.35	0.99	48.5	0.33	0.99	54.8
PRN 24	0.48	0.99	36.6	0.46	0.99	47.6	0.36	0.99	54.0
PRN 25	0.47	0.99	36.7	0.43	0.99	47.0	0.33	0.99	54.9
PRN 29	0.55	0.99	36.7	0.49	0.99	45.4	0.46	0.99	54.4

**Table 3:** Pseudorange measurement errors for PM and MPDR in the presence of chirp interference.

PRN	PM		MPDR	
	Mean (m)	Standard Deviation (m)	Mean (m)	Standard Deviation (m)
PRN 3	0.27	0.31	0.02	0.55
PRN 6	0.33	0.27	0.02	0.59
PRN 10	0.50	1.93	0.08	0.75
PRN 16	0.31	0.36	0.01	0.59
PRN 18	0.28	0.33	0.02	0.69
PRN 21	0.31	0.30	−0.04	0.58
PRN 24	0.35	0.49	0.02	0.65
PRN 25	0.38	0.55	0.04	0.55
PRN 29	0.31	0.40	−0.01	0.60

**Table 4:** Carrier phase measurement errors using PM and MPDR in the presence of chirp interference.

PRN	PM		MPDR	
	Mean (mm)	Standard Deviation (mm)	Mean (mm)	Standard Deviation (mm)
PRN 3	10.7	0.4	0.04	0.4
PRN 6	3.4	0.2	−0.01	0.4
PRN 10	30.5	1.8	0.07	0.7
PRN 16	5.0	0.4	0.05	0.6
PRN 18	7.6	0.3	−0.01	0.5
PRN 21	1.4	0.2	−0.05	0.5
PRN 24	−16.2	0.4	0.01	0.5
PRN 25	23.3	0.4	−0.03	0.5
PRN 29	−6.9	0.3	0.03	0.5

**Table 5:** Carrier phase position errors with PM and MPDR.

Position Errors	PM		MPDR	
	Mean	Standard Deviation	Mean	Standard Deviation
East error (cm)	3.2	1.1	−0.001	0.003
North error (cm)	10.6	3.3	0.002	0.005
Height error (cm)	−1.4	6.8	−0.005	0.005

**Table 6:** Tracking performance of different PRNs, PM and MPDR in the presence of chirp interference.

PRN	Single Antenna with Interference			PM			MPDR		
	RMSE Doppler (Hz)	PLI	C/N_0_ (dB-Hz)	RMSE Doppler (Hz)	PLI	C/N_0_ (dB-Hz)	RMSE Doppler (Hz)	PLI	C/N_0_ (dB-Hz)
PRN 1	0.98	0.98	34.1	0.43	0.99	44.9	0.66	0.99	46.5
PRN 4	0.97	0.98	36.7	0.45	0.99	44.7	0.49	0.99	48.1
PRN 11	0.91	0.97	37.6	0.45	0.99	46.8	0.31	0.99	49.1
PRN 13	0.89	0.97	37.5	0.45	0.99	48.2	0.28	0.99	50.9
PRN 15	0.92	0.97	33.8	0.85	0.99	43.4	0.64	0.99	48.6
PRN 17	0.92	0.97	38.2	0.28	0.99	45.5	0.47	0.99	47.7
PRN 28	0.92	0.97	38.7	0.23	0.99	47.7	0.34	0.99	49.8
PRN 30	0.98	0.99	41.1	0.60	0.99	44.7	0.29	0.99	52.7

**Table 7:** Pseudorange measurement errors for PM and MPDR in the presence of chirp interference.

PRN	PM		MPDR	
	Mean (m)	Standard Deviation (m)	Mean (m)	Standard Deviation (m)
PRN 1	−1.42	1.94	−1.71	2.92
PRN 4	0.34	1.32	0.26	2.40
PRN 11	0.22	1.49	1.27	2.42
PRN 13	0.48	1.33	0.47	2.38
PRN 15	−1.05	3.31	−0.55	3.21
PRN 17	−0.06	1.48	0.24	2.01
PRN 28	−0.01	0.69	0.29	1.11
PRN 30	−0.69	1.31	1.12	1.35

**Table 8:** Carrier phase measurement errors using PM and MPDR with chirp interference.

PRN	PM		MPDR	
	Mean (mm)	Standard Deviation (mm)	Mean (mm)	Standard Deviation (mm)
PRN 1	−7.3	2.0	0.3	1.6
PRN 4	7.1	1.7	0.9	2.0
PRN 11	−10.9	3.8	0.2	1.4
PRN 13	−5.0	1.3	−0.8	1.3
PRN 15	−2.3	1.8	1.0	1.7
PRN 17	8.2	1.9	0.5	2.1
PRN 28	1.6	0.8	0.9	1.1
PRN 30	2.3	1.1	0.5	1.5

**Table 9:** Carrier phase based position errors with actual data.

Position Errors	PM		MPDR	
	Mean	Standard Deviation	Mean	Standard Deviation
East error (cm)	−63.4	36.2	−0.04	0.08
North error (cm)	104.5	65.0	−0.25	0.06
Height error (cm)	−5.09	78.6	−0.06	0.16

## Appendix A

Tracking performance of different PRNs with CW and BWGN interference are provided in Table A1 and Table A2.

**Table A1:** Tracking performance of different PRNs in the presence of CW interference.

PRN	Single Antenna			PM			MPDR		
	RMSE Doppler (Hz)	Mean PLI	C/N_0_ (dB-Hz)	RMSE Doppler (Hz)	PLI	C/N_0_ (dB-Hz)	RMSE Doppler (Hz)	PLI	C/N_0_ (dB-Hz)
PRN 3	0.51	0.99	33.4	0.42	0.99	48.9	0.34	0.99	54.69
PRN 6	36.14	-	37.3	0.42	0.99	48.02	0.33	0.99	54.59
PRN 10	0.67	0.99	35.09	1.11	0.99	42.13	0.34	0.99	54.59
PRN 16	3.25	-	39.4	0.75	0.99	45.92	0.51	0.99	54.26
PRN 18	0.80	0.99	36.25	0.51	0.99	47.38	0.40	0.99	54.26
PRN 21	1.97	0.98	37.01	0.47	0.99	48.56	0.35	0.99	54.66
PRN 24	0.57	0.99	34.79	0.46	0.99	47.66	0.37	0.99	54.80
PRN 25	0.49	0.99	35.6	0.46	0.99	47.08	0.39	0.99	54.29
PRN 29	0.90	0.99	34.8	0.67	0.99	45.43	0.51	0.99	54.27

**Table A2:** Tracking performances of different PRNs in the presence of BWGN interference.

PRN	Single Antenna			PM			MPDR		
	RMSE Doppler (Hz)	PLI	C/N_0_ (dB-Hz)	RMSE Doppler (Hz)	PLI	C/N_0_ (dB-Hz)	RMSE Doppler (Hz)	PLI	C/N_0_ (dB-Hz)
PRN 3	1.10	0.98	37.7	0.32	0.99	48.9	0.26	0.99	54.4
PRN 6	1.10	0.98	37.7	0.36	0.99	48.0	0.29	0.99	54.3
PRN 10	1.03	0.98	37.7	-	-	-	0.35	0.99	54.0
PRN 16	1.14	0.98	37.7	0.46	0.99	45.9	0.39	0.99	54.0
PRN 18	1.08	0.98	37.7	0.39	0.99	47.3	0.29	0.99	54.0
PRN 21	1.10	0.98	37.7	0.35	0.99	48.5	0.33	0.99	54.3
PRN 24	1.15	0.98	37.7	0.37	0.99	47.6	0.28	0.99	54.5
PRN 25	1.09	0.98	37.7	0.40	0.99	47.0	0.25	0.99	54.3
PRN 29	1.17	0.98	37.7	0.50	0.99	45.4	0.46	0.99	54.0

## Appendix B

Measurement distortions in the presence of CW and BWGN interference are provided in this section.

**Table B1:** Pseudorange measurement errors for PM and MPDR in the presence of CW interference.

PRN	PM		MPDR	
	Mean (m)	Standard Deviation (m)	Mean (m)	Standard Deviation (m)
PRN 3	−0.11	0.33	−0.06	0.55
PRN 6	−0.13	0.29	0.01	0.62
PRN 10	−0.19	1.73	0.05	0.80
PRN 16	−0.11	0.37	−0.06	0.63
PRN 18	−0.14	0.34	0.03	0.65
PRN 21	−0.09	0.25	−0.01	0.57
PRN 24	0.01	0.49	0.05	0.70
PRN 25	0.07	0.52	0.03	0.61
PRN 29	−0.06	0.43	0.03	0.69

**Table B2:** Carrier phase measurement errors using PM and MPDR with CW interference.

PRN	PM		MPDR	
	Mean (mm)	Standard Deviation (mm)	Mean (mm)	Standard Deviation (mm)
PRN 3	10.9	0.4	0.01	0.4
PRN 6	3.4	0.3	−0.01	0.4
PRN 10	29.6	1.5	0.07	0.7
PRN 16	4.8	0.4	0.05	0.6
PRN 18	7.5	0.3	−0.01	0.5
PRN 21	1.5	0.2	−0.05	0.5
PRN 24	−16.0	0.3	0.01	0.5
PRN 25	23.1	0.5	−0.03	0.5
PRN 29	−6.7	0.4	0.03	0.5

**Table B3:** Pseudorange measurement errors for PM and MPDR in the presence of BWGN interference.

PRN	PM		MPDR	
	Mean (m)	Standard Deviation (m)	Mean (m)	Standard Deviation (m)
PRN 3	−0.11	0.34	−0.06	0.55
PRN 6	−0.13	0.28	0.01	0.62
PRN 10	-	-	0.05	0.81
PRN 16	−0.10	0.37	−0.06	0.63
PRN 18	−0.14	0.36	0.03	0.65
PRN 21	−0.09	0.25	0.01	0.57
PRN 24	0.01	0.49	0.05	0.70
PRN 25	0.07	0.53	−0.04	0.61
PRN 29	−0.06	0.45	0.04	0.69

**Table B4:** Carrier phase measurement errors using PM and MPDR with BWGN interference.

PRN	PM		MPDR	
	Mean (mm)	Standard Deviation (mm)	Mean (mm)	Standard Deviation (mm)
PRN 3	10.9	0.4	0.01	0.5
PRN 6	3.4	0.3	−0.01	0.5
PRN 10	-	-	0.07	0.7
PRN 16	4.8	0.4	0.05	0.6
PRN 18	7.5	0.3	−0.01	0.5
PRN 21	1.5	0.2	−0.05	0.5
PRN 24	−16.0	0.4	0.01	0.5
PRN 25	23.1	0.5	−0.03	0.5
PRN 29	−6.7	0.4	0.03	0.5
